# Assessment of the effect of agro-industrial by-products rich in polyphenols on *in vitro* fermentation and methane reduction in sheep

**DOI:** 10.3389/fvets.2025.1530419

**Published:** 2025-01-30

**Authors:** Alessandro Vastolo, Blandine Mora, Dieu donné Kiatti, Martina Nocerino, Serkos Haroutounian, Rania D. Baka, Panagiota Ligda, Monica Isabella Cutrignelli, Vincent Niderkorn, Serena Calabrò

**Affiliations:** ^1^Department of Veterinary Medicine and Animal Production, University of Napoli Federico II, Naples, Italy; ^2^NRAE, Université Clermont Auvergne, VetAgro Sup, UMR Herbivores, Saint-Genès-Champanelle, France; ^3^Department of Animal Science, School of Animal Biosciences, Agricultural University of Athens, Athens, Greece; ^4^Veterinary Research Institute, Hellenic Agricultural Organization (ELGO) – DIMITRA, Thessaloniki, Greece; ^5^Department of Animal Nutrition and Feed Technology, Faculty of Animal Husbandry, Universitas Padjadjaran, Jatinangor, Indonesia

**Keywords:** environmental impact, *in vitro* fermentation, methane, polyphenols, tannins

## Abstract

**Introduction:**

This study aimed to evaluate, using the *in vitro* gas production technique, the effect of including eight agro-industrial by-products (carob, grape, two types of olive pomace, citrus pulp, tomato, and hazelnut skin) on fermentation end-products, ruminal degradability, and methane production in sheep diets.

**Methods:**

The by-products were included at 10% dry matter in the control (CTR) diet, commonly adopted for adult sheep (80% natural grassland and 20% concentrate), and incubated at 39°C under anaerobic conditions.

**Result and discussion:**

After 24 h of the incubation, the organic matter degradability (OMD24h) and methane production were assessed. After 120 h of the incubation, the organic matter degradability (OMD120h), volume of gas produced (OMCV), fermentation kinetics, pH, volatile fatty acids (VFAs), and ammonia were evaluated. Dunnett’s test was used to compare the differences between the control and experimental diets, and multivariate analysis was performed to highlight the differences among the diets based on their *in vitro* characteristics. The results indicated that the inclusion of the by-products decreased the degradability and increased gas production after 120 h of the incubation. The by-products from the hazelnuts, citrus, grapes, and tomatoes significantly (*p* < 0.001) reduced the methane production, whereas the pomegranate, grape, 3-phase olive cake, tomato, and hazelnut by-products significantly (*p* < 0.001) increased the acetate production. The multivariate analysis showed that the butyrate concentration was a determining factor in the differences between the diets. The concentration of polyphenols in the selected agro-industrial by-products could modify fermentation parameters and metabolic pathways, leading to reduced methane production.

## 1 Introduction

According to the European Commission (1), the term “by-product” refers to any substance or object that results from a production process and whose existence is not intended in the primary process target (2). The volume of by-products, mainly originating from industrial processes, is constantly growing globally every year. In this regard, the largest proportion of residues (approximately 40–50% of total discards) consists of fruit and vegetable by-products (3). A total of 88 million tons (±14Mt) of food waste are produced along the supply chain in the European Union (EU). On a global scale, food losses and waste account for approximately 1.3 billion tons per year, or 16% of the total food supply. In the case of fruits and vegetables, food losses are in the range of 20–40%, beginning in initial agricultural production and continuing throughout processing, up to the final consumer (3, 4). This waste results in the loss of resources along the supply chain, such as water, land, and energy, and has a significant environmental impact (5–7). Considering the volatility of feed raw material prices, it is necessary to find alternative feeding options (8–10). By-products, particularly fruit and vegetable wastes, could serve as a feed resource rich in high-value nutrients for livestock.

Fruit and vegetable by-products, rich in tannins and flavonoids, may exhibit antimicrobial, antiparasitic, and antioxidant activity and could decrease methane and ammonia emissions, thereby reducing environmental impact (11–13). Indeed, *in vitro* trials (14–16) have demonstrated that some by-products, such as grape pomace and olive cake, could affect fermentation parameters and decrease methane emissions because of the presence of valuable bioactive molecules (17–19). Although by-products have long been included in the diets of livestock, providing added value to animal health and production (19), several issues, such as storage, seasonality, and variability in chemical composition (20, 21), make their inclusion in animal diet challenging (22).

Further studies are needed to gain a better understanding and characterization of the nutritional qualities of by-products. Therefore, the objective of this study was to evaluate, using the *in vitro* gas production technique, the effect of including eight agro-industrial by-products (carob, grape, two types of olive pomace, citrus pulp, tomato, and hazelnut skin) on fermentation end-products, ruminal degradability, and methane production in sheep diets.

## 2 Materials and methods

### 2.1 Chemical composition and bioactive compounds

The eight agro-industrial by-products (Table 1) were selected for their local availability in France, Italy, and Greece and were derived from different food industrial processing methods. In this study, two different types of olives were tested because a two-phase olive cake (OC2) by-product has higher moisture and lower fat content compared to a three-phase olive cake (OC3) and is derived from a more resourceful and environmentally friendly centrifugation process (23). The grape extract was obtained after the mechanical pressing of grapes to concentrate the polyphenols. Since bioactive compounds are very sensitive to high temperatures, all by-products were dried at 40°C for 3–4 d. All samples were milled (1.1 mm) and analyzed for dry matter (DM), crude protein (CP), ether extract (EE), and sugar contents (24). According to Van Soest et al. (25), the structural carbohydrate content (neutral detergent fiber, NDF; acid detergent fiber, ADF; and acid detergent lignin, ADL) was also determined, excluding the ash content. The total phenolic content (TPC), total flavonoid content (TFC), and total tannin content (TTC) were also reported. The TPC of all samples was estimated using the spectrophotometric method (26), the TFC was estimated by modifying the aluminum chloride method of Pekal and Pyrzynska (27), and the TTC of the methanolic extracts was determined using a modified version of the spectrophotometric method (Table 2) (26).

**Table 1:** Description and origin of the selected by-products.

Fruits	Family	Species	By-products	Origin
Citrus	*Rutaceae*	*Citrus senensis*	Pulp and peel	Italy
Olive	*Oleaceae*	*Olea europaea*	Cake (2-phase)	Italy
Hazelnuts	*Betulaceae*	*Corylysavellana*	Skin	Italy
Tomato	*Solanaceae*	*Solanum lycopersicum*	Skin	Italy
Carob	*Fabaceae*	*Ceratonia siliqua*	Pulp	Greece
Olive	*Oleaceae*	*Olea europaea*	Cake (3-phase)	Greece
Pomegranate	*Lythraceae*	*Punica granatum*	Peel and seeds	Greece
Grape	*Vitacea*	*Vitis vinifera*	Extract	France

**Table 2:** Proximate chemical composition and total content of the polyphenols, phenols, and tannins of the selected by-products.

By-products	DM	Ash	CP	EE	NDF	ADF	ADL	NSC	Sugar	TPC	TFC	TTC
	% as fed								% glucose	mg/GAE/g	mg QE/g	mg CE/g
Citrus	44.8	4.50	8.37	1.55	16.6	11.0	0.80	69.0	7.43	109	10.2	23.2
Olive cake (3-phase)	66.5	5.39	9.18	19.7	53.2	36.6	17.2	12.5	1.36	81.3	11.9	12.1
Hazelnuts	95.9	3.22	10.8	20.9	51.8	45.4	32.1	13.3	4.06	768	31.0	692
Tomato	17.0	6.24	20.7	9.41	57.8	45.4	25.0	5.87	0.12	76.4	5.41	13.7
Carob	88.8	3.15	6.33	0.67	29.0	25.2	13.6	60.9	7.06	71.1	5.66	23.6
Olive cake (2-phase)	50.6	5.34	10.9	14.4	51.4	37.2	22.2	17.9	0.37	334	<LOD	24.2
Pomegranate	27.0	5.92	4.25	0.82	28.2	19.8	5.60	60.8	7.66	nd	nd	nd
Grape	33.5	1.05	3.67	0.57	8.00	4.00	2.00	86.7	6.96	732	17.36	713

DM, dry matter; CP, crude protein; EE, ether extract; GAE, Gallic Acid Equivalents; QE, Quercetin Equivalents; CE, Catechin Equivalents. NDF, neutral detergent fiber; ADF, acid detergent fiber; ADL, acid detergent lignin; NSC, non-structural carbohydrates (=100 – CP– EE– NDF– Ash). TPC, total polyphenol content; TFC, total phenolic content; TTC, total tannin content; nd, not detected; LOD, limit of detectability.

### 2.2 *In vitro* gas production

The *in vitro* experimental design included a control (CTR) and seven experimental diets for adult sheep.

All diets consisted of 80% natural grassland and 20% concentrate (ingredients: soybean meal corn meal, wheat bran, and vitamin and minerals supplementation). Each by-product was included in an experimental diet at 10% on a concentrate DM basis. The dose was defined to exhibit the potential maximum effect of the by-products in the diet on ruminal fermentation. The diets were formulated to guarantee the following nutritional characteristics: NDF 42.8 ± 0.35% DM and CP 20.8 ± 0.38% DM.

All diets were incubated in serum flasks (one run, six replications per substrate, *n* = 48; mean weight: 1.0025 ± 0.00010 g) with pooled buffered sheep rumen liquor (10 mL) at 39°C under anaerobic conditions (28, 29). The rumen liquor was collected at the slaughterhouse from three healthy grazing adult sheep (age: 18–20 months; weight 45–50 kg). The rumen fluid was immediately stored in a pre-heated thermos and transported to the Feed Evaluation laboratory at the Department of Veterinary Medicine and Animal Production (University of Napoli Federico II) within 2 hours. In the laboratory, the rumen fluid was pooled to limit the donor effect, mixed, strained through four layers of cheesecloth, and diluted in a buffered medium (75 mL,1:7.5 rumen liquor:medium ratio). A reducing agent (4 mL) for oxidation was added to the flasks. In three bottles, the incubation lasted 120 h, and the produced gas was recorded 21 times (at intervals of 2 to 24 h) using a manual pressure transducer (Cole and Palmer Instrument Co, Vernon Hills, IL, United States). The cumulative volume of the gas produced was related to the incubated and degraded organic matter (OMCV and Yield, respectively, mL/g). After the incubation, the residue in each serum flask was filtered through crucibles (porosity #2) and burned in a muffle furnace at 550°C for 3 h to assess the organic matter degradability (OMD120h, %), determined by the weight difference between the empty crucible and the crucible after ashing.

### 2.3 Methane production assessment

The three flasks from the six replications of each diet were removed at 24 h for the methane (CH_4_) and organic matter degradability (OMD24h) assessment. Three mL of the gas phase was sampled in duplicate from each serum flask using a gastight syringe and injected into a gas chromatograph (ThermoQuest 8000top Italia SpA, Rodano, Milan, Italy), equipped with a loop TC detector and a packed column (HaySepQ SUPELCO, 3/16-inch, 80/100 mesh) (30). The methane production was reported as a function of the incubated organic matter (CH_4_iOM) and organic matter degradability (CH_4_dOM).

### 2.4 *In vitro* fermentation end-products

At the end of the incubation period, the pH of the fermentation liquor was measured with a pH meter (ThermoOrion 720 A+, Fort Collins, CO, United States). The fermentation liquor (5 mL) of each serum flask was collected and centrifuged at 12,000 (x) g for 10 min at 4°C (Universal 32R centrifuge, Hettich FurnTech Division DIY, Melle-Neuenkirchen, Germany). Subsequently, 1 mL of the supernatant was mixed with 1 mL of oxalic acid (0.06 Mol). The volatile fatty acids (VFAs) were measured using gas chromatography (ThermoQuest 8000top Italia SpA, Rodano, Milan, Italy; fused silica capillary column 30 m, 0.25 mm ID, 0.25 μm film thickness). An external standard mixture consisting of acetic, propionic, butyric, iso-butyric, valeric, and isovaleric acids was used. The branched-chain fatty acids (BCFAs) proportion was calculated as follows: (Iso-Butyrate + Iso-Valerate)/total VFA. Ammonia was analyzed by spectrophotometric analysis (340 nm) using the Enzytec assay kit (art. n° E8390, R-Biopharm AG, Darmstadt, Germany).

### 2.5 Data processing and statistical analysis

For fermentation kinetics estimation, the gas production data were fitted to the sigmoidal model for each bottle (31):


$$G=A/\left(1+{\left(B/t\right)}^{C}\right)$$

where G is the total gas produced (mL/g incubated OM) at time (t), A refers to the asymptotic gas production (mL/g), B is the time at which half of A is reached (h), and C is the curve switch.

The maximum fermentation rate (R_max_, mL/h) and the time at which it occurred (T_max_, h) were determined using model parameters (32):


$$R_{\text{max}}=\frac{\left(A\;x\;B^{C}\right)\;x\;B\;x\;{T_{\text{max}}}^{\left(B-1\right)}}{\left(1+C^{B}\right)\;x\;{\left(T_{\text{max}}-B\right)}^{2}}$$

$$T_{\text{max}}=C\;x\;\left(B–1\right)/{\left(B+1\right)}^{1/B}$$

Statistical analyses for the *in vitro* fermentation parameters (OMD, OMCV, and Yield), kinetics (T_max_, R_max_), end-products (pH, VFAs, and BCFAs), and OMD and CH_4_ measured at 24 h were performed using one-way ANOVA (JMP®, Version 14 SW, SAS Institute Inc., Cary, NC, United States, 1989–2019) to evaluate the effect of the substrates as a fixed factor. The significance level was verified using Tukey’s HSD test with *p*-values <0.01 and < 0.05. Dunnett’s test was performed to observe the differences between the control and experimental diets. The Shapiro–Wilk test was performed for the normally distributed data. A stepwise discriminant analysis (STEPDISC, JMP software) was applied to the entire set of variables to select those that best discriminated between the diets. Afterward, the selected variables were used in canonical discriminant analysis (CANDISC procedure), a dimension reduction approach to derive canonical functions and summarize the variation among groups.

## 3 Results

### 3.1 *In vitro* parameters and fermentation kinetics

**Figure 1:**
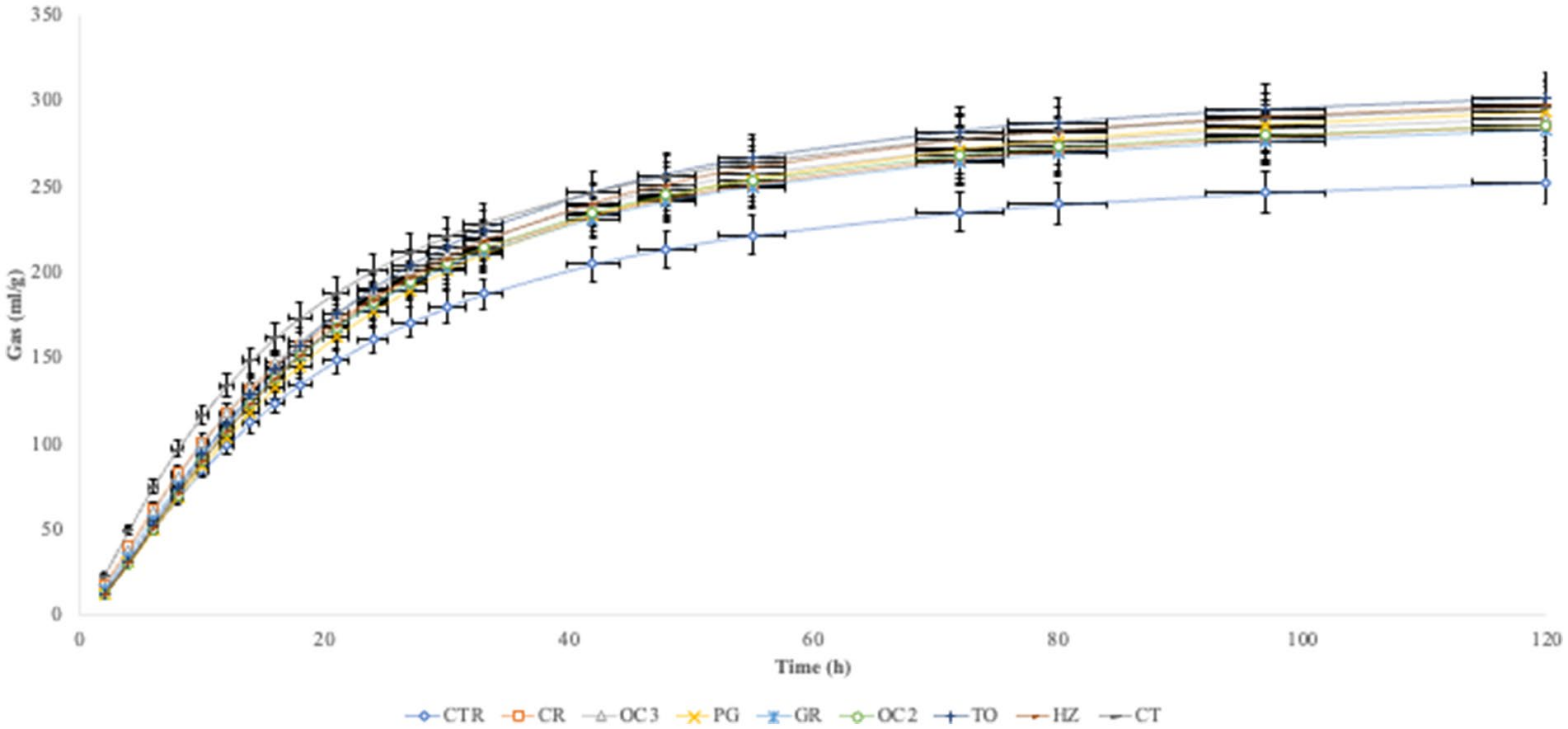
*In vitro* gas production over time.

**Figure 2:**
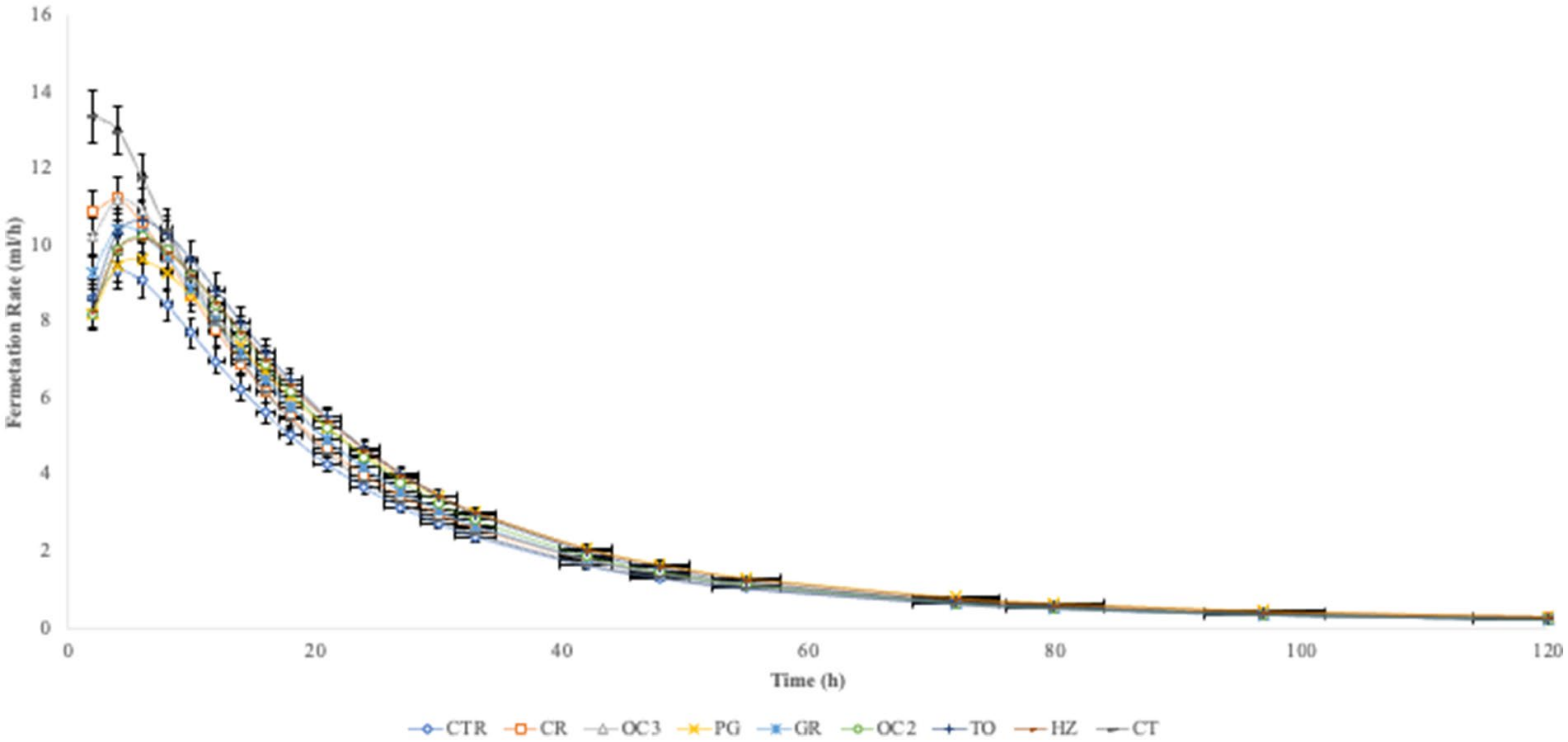
*In vitro* fermentation kinetic over time.

In Table 3, the *in vitro* parameters are presented. In all experimental diets, the addition of the by-products to the control diet significantly decreased (*p* < 0.05) the organic matter degradability (OMD), particularly when the olive cake from Italy (OC2) was included, followed by the pomegranate (PG). On the contrary, the inclusion of the by-products in the control diet significantly increased the gas production (OMCV and Yield) in all experimental diets during the first 6 h of the incubation (Figure 1). Regarding the fermentation kinetics (Figure 2), the pomegranate (PG), grape pomace (GR), olive cake from Greece (OC3), tomato (TO), and hazelnut (HZ) by-products significantly increased (*p* < 0.001) the time to the maximum fermentation rate (T_max_) of the diet, while the citrus (CT) by-product supplementation to the control diet significantly decreased (*p* < 0.001) the T_max_ value. Apart from the PG diet, all other experimental diets, especially the one with the citrus (CT) by-products, showed a significant increase (*p* < 0.01) in the fermentation rate (R_max_).

**Table 3:** *In vitro* organic matter degradability, gas production, and fermentation rate of the control and experimental diets.

Items	OMD120h	OMCV	Yield	T_max_	R_max_
	%	mL/g	mL/g	h	mL/h
CTR	81.1	257	314	3.77	9.37
CR	75.2 ***	285 ***	369 **	3.65 NS	11.3 ***
OC2	53.3 ***	294 ***	545 ***	4.60 NS	11.2 ***
PG	77.7 *	283 ***	364 ***	5.38 ***	9.33 NS
GR	73.4 ***	285 ***	387 ***	4.99 **	10.5 ***
OC3	72.2 ***	295 ***	419 ***	5.77 ***	10.2 ***
TO	72.2 ***	297 ***	411 ***	5.75 ***	10.6 ***
HZ	73.2 ***	294 ***	403 ***	6.58 ***	10.5 ***
CT	67.6 ***	300 ***	438 ***	2.03 ***	14.5 ***
MSE	1.11	8.34	12.7	0.233	0.05

CTR, control diet; CR, control diet + carob; OC2, control diet + Olive cake from Italy; PG, control diet + pomegranate; GR, control diet + grape; OC3, control diet + Olive cake from Greece; TO, control diet + tomato; HZ, control diet + hazelnuts; CT, control diet + citrus. OMD120h, degraded organic matter after 120 h of the incubation; OMCV, cumulative volume of gas related to incubated OM; Yield, cumulative volume of gas related to degraded OM; T_max_, maximum time which occurs R_max_, maximum fermentation rate. *, **, ***: *p* < 0.05, 0.01, 0.001, respectively; NS, not significant; MSE, mean square error.

### 3.2 *In vitro* fermentation end-products

In Table 4, the end-products of the *in vitro* fermentation are reported. All experimental diets had significantly (*p* < 0.001) higher pH levels compared to the control diet. The addition of the OC3 and GR to a standard diet significantly decreased (*p* < 0.05) the ammonia production. The inclusion of the by-products to the control diet significantly decreased (*p* < 0.001) the production of the VFAs. All by-products, except for the OC2, significantly decreased (*p* < 0.0001) the BCFA production. Similarly, the inclusion of all by-products significantly decreased (*p* < 0.001) the propionate production, except for the CT and PG by-products. In contrast, the PG, GR, OC3, TO, and HZ diets significantly increased (*p* < 0.0001) the acetate production. The diets including the OC3, TO, and HZ demonstrated a lower percentage of the iso-butyrate compared to the CTR diet. Regarding the percentage of the butyrate, except for the CR and CT, the inclusion of all other by-products (i.e., PG, GR, OC2, TO, and HZ) in the control diet, significantly decreased (*p* < 0.0001) its production. Similarly, except for the OC2, the inclusion of the by-products in the control diet significantly decreased (*p* < 0.0001) the iso-valerate percentage. The inclusion of the CR, OC2 from Italy, PG, GP, and TO by-products in the control diet significantly increased (*p* < 0.0001) the production of the valerate. The carob, GR, OC3, TO, and HZ diets significantly increased (*p* < 0.0001) the acetate/propionate ratio.

**Table 4:** *In vitro* fermentation end-products of the control and experimental diets.

Diet	pH	NH_3_	VFA	BCFA	Ace	Prop	Iso-but	But	Iso-val	Val	Ace/Prop
		mmol/l		% VFA							
CTR	6.36	8.69	64.6	5.58	59.4	19.9	2.26	13.1	3.44	2.17	2.91
CR	6.41 ***	7.85 NS	54.3 ***	5.33 *	59.6 NS	18.2 ***	2.14 NS	14.2 ***	3.23 *	2.50 ***	3.37 **
OC2	6.47 ***	9.41 NS	56.8 ***	5.70 NS	59.7 NS	19.2 *	2.23 NS	13.0 NS	3.47 NS	2.35 ***	3.11 NS
PG	6.41 ***	7.19 NS	55.8 ***	4.98 ***	60.7 **	20.3 NS	1.99 NS	11.5 ***	3.01 ***	2.38 ***	3.01 NS
GR	6.42 ***	6.54 *	56.5 ***	5.15 ***	62.9 ***	17.4 ***	1.86 NS	12.1 ***	3.16 ***	2.37 ***	3.62 ***
OC3	6.46 ***	6.71 *	59.2 ***	4.46 ***	63.4 ***	18.6 ***	1.78 **	11.7 ***	2.67 ***	2.10 NS	3.47 ***
TO	6.45 ***	8.29 NS	60.3 ***	4.71 ***	64.2 ***	17.8 ***	1.83 *	10.8 ***	2.91 ***	2.47 ***	3.59 ***
HZ	6.41 ***	8.24 NS	57.6 ***	4.41 ***	62.7 ***	18.3 ***	1.81 *	12.4 ***	2.67 ***	2.21 NS	3.50 ***
CT	6.43 ***	8.00 NS	57.8 ***	5.14 ***	59.0 NS	19.3 NS	2.02 NS	14.1 ***	3.08 ***	2.24 NS	3.07 NS
MSE	42e-5	0.782	0.50	0.008	0.15	0.05	0.02	0.008	0.003	0.002	0.01

CTR, control diet; CR, control diet + carob; OC2, control diet + OC2 by-products; PG, control diet + pomegranate; GR, control diet + grape; OC3, control diet + OC3by-products; TO, control diet + tomato; HZ, control diet + hazelnuts; CT, control diet + citrus; NH_3_, ammonia; VFA, volatile fatty acids; BCFA, branched-chain fatty acids; Ace, acetate; Prop, propionate; Iso-but, Iso-butyrate; But, butyrate; Iso-val, Iso-valerate; Val, valerate; Ace/Prop, acetate/propionate ratio. *, **, ***: *p* < 0.05, 0.01, 0.001, respectively; NS, not significant; MSE, means square error.

### 3.3 *In vitro* fermentation parameters

The *in vitro* parameters after 24 h of the incubation are presented in Table 5. Regarding the organic matter degradability (OMD24h), the inclusion of the olive cakes (OC2 and OC3), TO, and HZ decreased the values compared to the CTR diet. Few effects were observed on the methane production when expressed in ml/g iOM. Only the supplementation of the HZ and CT by-products to the control diet significantly decreased the methane production in terms of mL/giOM. The by-products of the HZ, CT, GR, and TO significantly decreased (*p* < 0.01) the methane production when related to the organic matter degraded (CH4dOM). On the contrary, the inclusion of the olive cakes in the control diet significantly increased (*p* < 0.001) the methane production when reported as mL/OMD.

**Table 5:** *In vitro* organic matter degradability and methane production after 24 h of the incubation.

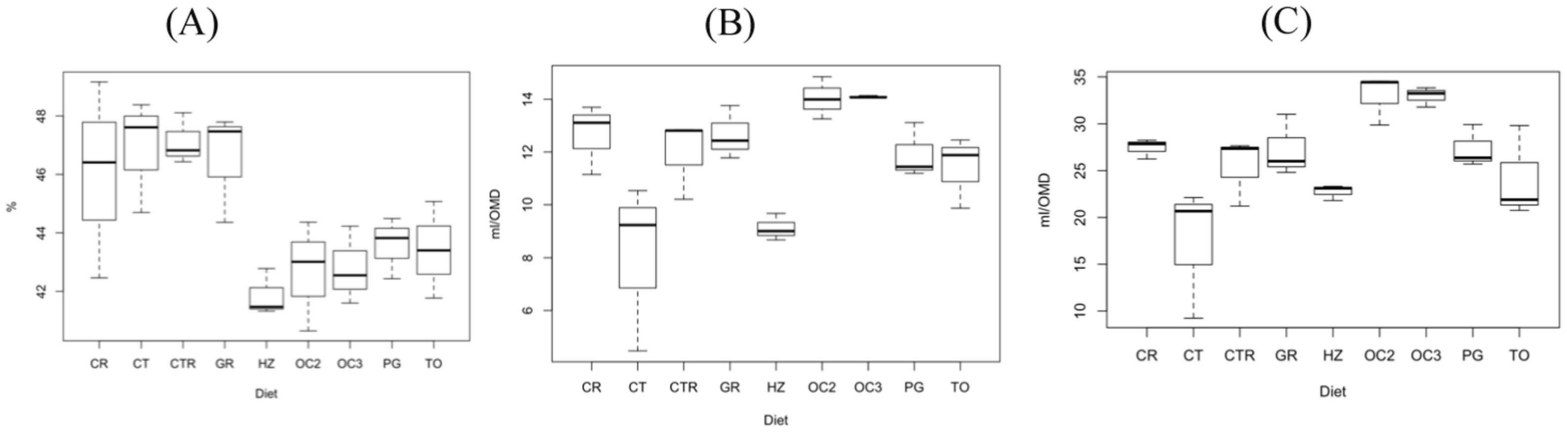			
Diet	OMD24h	CH_4_iOM	CH_4_dOM
	%	mL/giOM	mL/OMD
CTR vs.			
CR	NS	NS	NS
OC2	*	NS	***
PG	*	NS	NS
GR	NS	NS	**
OC3	*	NS	***
TO	*	NS	***
HZ	**	***	***
CT	NS	**	***

*In vitro* organic matter degradability **(A)** and methane production by incubated **(B)** and degraded **(C)** organic matter after 24 h of incubation. CTR, control diet; CR, control diet + carob; OC2, control diet + OC2 by-products; PG, control diet + pomegranate; GR, control diet + grape; OC3, control diet + OC3 by-products; TO, control diet + tomato; HZ, control diet + hazelnuts; CT, control diet + citrus; OMD 24 h, degraded organic matter after 24 h of the incubation; CH_4_iOM, methane related to incubated organic matter; CH_4_dOM, methane related to degraded organic matter at 24 h of the incubation. *, **, ***: *p* < 0.05, 0.01, 0.001, respectively; NS, not significant; MSE, mean square error.

### 3.4 Multivariate analysis

**Figure 3:**
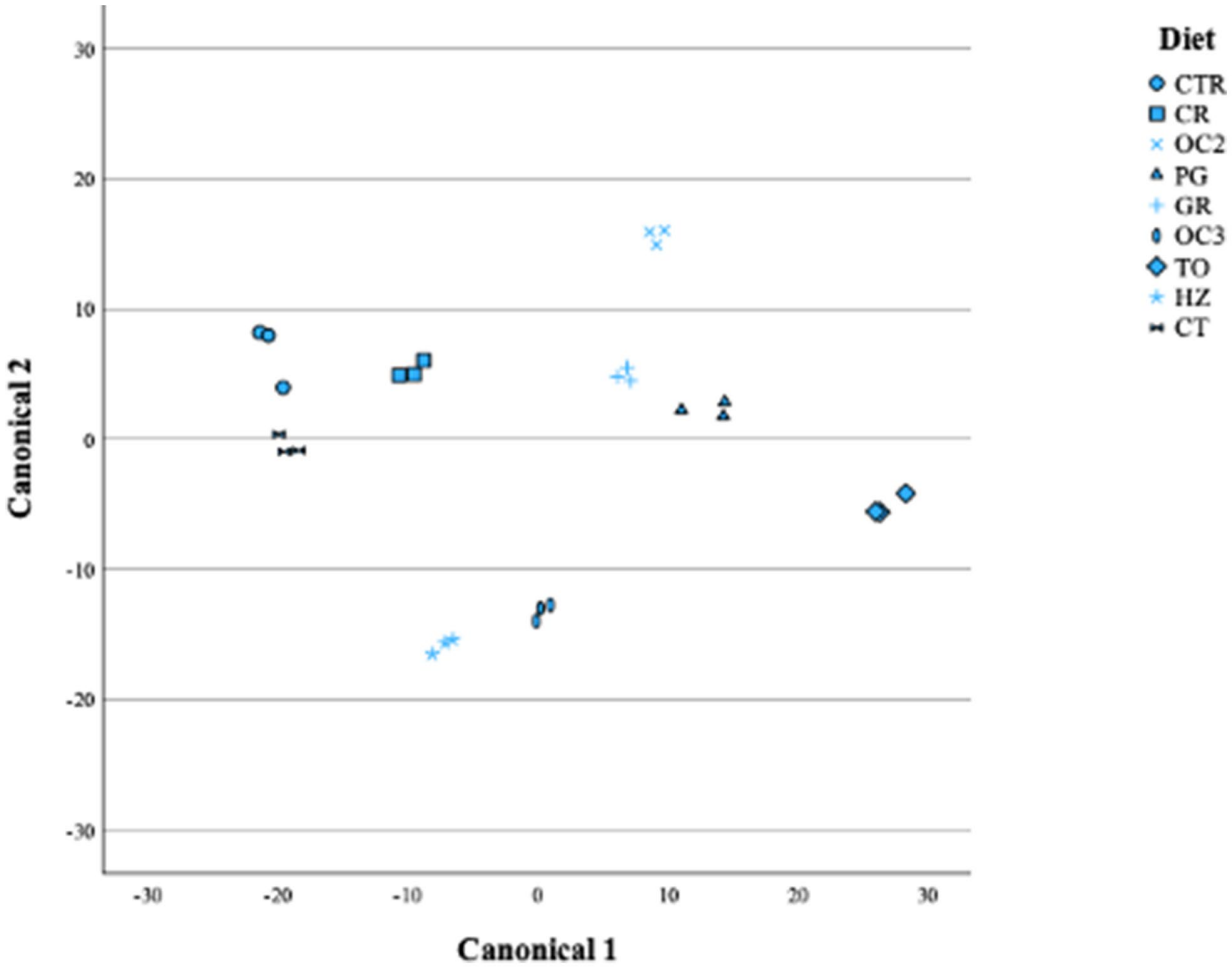
Plot of canonical 1 (Can 1) and canonical 2 (Can 2).

Table 6 shows the canonical structure; the first canonical variable explained more than 70% of the total variability, while the second explained less than 20%. As evidenced by the distribution of the diets in Figure 3, the first canonical variable was positively correlated with the OMD, Rmax, VFAs, BCFAs, and propionic, butyric, and iso-valerianic acids and was negatively correlated with the cellulose, OMCV, Tmax, methane production, and acetic and valerianic acids. The second canonical variable was positively correlated with the Rmax, methane production, BCFAs, propionate, butyrate, iso-valerate, and valerate and negatively correlated with the OMD, OMCV, Tmax, volatile fatty acids, and acetate.

**Table 6:** Total canonical structure: correlations between the canonical variables and original variables.

Parameter	Can 1	Can 2
OMD120h	0.249	−0.419
OMCV	−0.445	−0.112
T_max_	−0.609	−0.490
R_max_	0.262	0.244
CH_4_iOM	−0.310	0.338
CH_4_dOM	−0.327	0.244
VFA	0.242	−0.319
BCFA	0.300	0.852
Ace	−0.631	−0.541
Prop	0.217	0.151
But	0.837	0.387
Iso-val	0.315	0.846
Val	−0.404	0.596
Variance explained (%)	71.7	19.1

Can 1, Canonical 1; Can 2, Canonical 2; OMD, degraded organic matter at 120 h of the incubation; OMCV, cumulative volume of gas related to incubated OM; T_max_, time at which the maximum fermentation rate occurred; R_max_, maximum fermentation rate; CH_4_iOM, methane related to incubated organic matter; CH_4_dOM, methane related to degraded organic matter at 24 h of the incubation; VFA, volatile fatty acids; BCFA, branched-chain fatty acids; Ace, acetate; Prop, propionate; Iso-but, Iso-butyrate; But, butyrate; Iso-val, Iso-valerate; Val, valerate.

## 4 Discussion

The inclusion of the selected by-products in the diet, at a level of 10% DM, affected the fermentation parameters during the incubation (120 h). In particular, the experimental diets showed a reduction in the organic matter degradability and an increase in the gas production (OMCV and Yield). The chemical composition of the selected by-products likely contributed to these results. The high content of the lipids of some by-products, such as the olive cake and hazelnut skin, contributed to the reduced diet digestibility (33). Furthermore, the majority of the by-products reported high lignin content, which is a highly resistant compound that is only partially degraded by the microbial population in the rumen. However, lignin content is not directly responsible for diet digestibility; its association with other chemical components can influence the properties of fermentation, including the enzymatic degradation of structural carbohydrates (34). Indeed, by-products rich in phenolic compounds, such as hazelnut skin, grape pomace, and olive cake, could limit cellulolytic and fibrolytic microbial activity due to the formation of complexes with lignocellulose, which reduce fiber degradability (35). A previous *in vitro* study (36) showed that high content of condensed tannins bound proteins and reduced organic matter degradation. Moreover, tannins have a protein-binding property that leads to a reduction in dietary protein degradation by the proteolytic microbial population, limiting ammonia concentration (37). Notwithstanding the reduction in the digestibility, the cumulative gas production was higher in all samples compared to the control diet. The fermentation rate exhibited a similar trend, except for the PG diet. These results can be attributed to the presence of non-structural carbohydrates (38).

The variation in terms of the fermentation and gas production affected the pH level in the fermentation liquor at the end of the incubation, which was within normal values for the ruminants, ranging between 6.41 and 6.47 across all tested diets (39). The inclusion of the by-products in the diets did not affect the ammonia production, except for the GP and OC3 diets, in which it decreased the ammonia content and reduced the total VFA production. As previously reported, these results could be explained by the high content of polyphenols and tannins in these by-products, which could bind nutrients, such as protein and carbohydrates, leading to a reduction in fermentation products in the rumen (40).

The inclusion of agro-industrial by-products may lead to a shift in the metabolic pathways during the process of ruminal fermentation and the production of volatile fatty acids. Indeed, the GP, GR, OC3, TO, and HZ by-products increased the acetate levels in the diets compared to the propionate and butyrate. The decrease in the short-chain branched acids (iso-valerate, iso-butyrate, and BCFAs), which are end-products of protein metabolism, may be explained by the low protein content of the evaluated by-products and their high content of phenolic compounds (30).

Regarding the parameters obtained after 24 h of the incubation, the addition of the by-products to the control diet did not affect the organic matter degradability, except for the tomato, both olive cakes, and hazelnuts. The olive cakes demonstrated low *in vitro* degradability, which was also reported by several authors (23–41) and can be attributed to their chemical composition (high content of structural carbohydrates and lignin). Moreover, both OC2 and OC3 increased the methane production per gram of the OMD, with similar findings previously recorded by Marcos et al. (42), who observed an increasing trend in methane production when an exhausted olive cake was evaluated. On the contrary, most of the experimental diets showed lower methane production. When the methane production was related to the incubated organic matter (CH_4_iOM), only the HZ and CT diets showed significant differences compared to the CTR diet. Tannins may exhibit a modulatory action on microbial populations, especially affecting archaea and protozoa, which have been correlated with methane production in the rumen (43–48).

Niderkorn et al. (49) evaluated *in vitro* rumen fermentation parameters in diets including sainfoin (*Onobrychis viciifolia* Scop.) pellets and/or hazelnut (*Corylus avellana* L.) pericarps using a batch culture system for 24 h. The authors concluded that the inclusion of the sainfoin pellets and hazelnut pericarps in a basal diet resulted in lower rumen fermentability and that condensed tannins decreased methane production and protein degradability. Atalay et al. (16) recorded a low methanogenic potential of grape pomace. In this regard, published data have reported different results regarding the potential of by-products for methane mitigation. These discrepancies could be explained by several factors, such as the industrial process (50).

The current results obtained through a stepwise multivariate discriminant analysis indicated that eight different canonical variables emerged, but only two completely explained the variance. Furthermore, most of the variance was explained by canonical 1 (Table 6), with the butyrate being the most discriminant parameter (showing the highest positive correlation). This result was also confirmed by the Mahalanobis distance (data not shown), with the CTR and TO diets showing the greatest distance (819, *p* < 0.001). In this regard, most of the experimental diets, particularly the TO diet, showed a decrease in the butyric acid production. This *in vitro* result could be promising for formulating a diet that prevents metabolic disorders. Indeed, increases in butyric and propionic acids could lead to metabolic disorders, such as subacute acidosis (SARA). Volatile fatty acids are the modulators of the inflammatory response as they can activate neutrophils, which are essential for host defense. Butyric acid decreases several neutrophil functions, such as phagocytosis (51). Moreover, *β*-hydroxybutyric acid (BHBA) is a metabolite of butyrate metabolism, normally used to monitor and prevent ketosis (52).

## 5 Conclusion

The obtained *in vitro* results demonstrated that the addition of the agro-industrial by-products at 10% DM affected the fermentation parameters (organic matter degradability and gas production). The addition of these by-products in a diet composed of natural grassland and concentrate promoted a reduction in the methane production during the first 24 h of the fermentation and increased the acetic acid production, which serves as a source of energy for ruminants. Further studies should be conducted to determine the appropriate inclusion dose of agro-industrial by-products in the basal diet of ruminants to avoid adverse effects on rumen fermentation.

## Data Availability

The raw data supporting the conclusions of this article will be made available by the authors, without undue reservation.
