# Supplementary material for: Assessment of the effect of agro-industrial by-products rich in polyphenols on in vitro fermentation and methane reduction in sheep
Source: Front Vet Sci. 2025 Jan 30;12:1530419. doi: 10.3389/fvets.2025.1530419 (PMC11821959; doi:10.3389/fvets.2025.1530419)
Supplement: Supplementary file 1 [file Table_1.DOCX]

Supplementary Material

Canonical discriminant analysis: Mahalanobis quadratic distances.

|  | CTR | CR | OC2 | PG | GR | OC3 | TO | HZ | CT |
| --- | --- | --- | --- | --- | --- | --- | --- | --- | --- |
| CTR | - | 235 (NS) | 392  (*) | 463  (*) | 321  (*) | 437  (*) | 819  (***) | 423  (*) | 180  (NS) |
| CR |  | - | 266  (NS) | 376  (*) | 161  (NS) | 452  (*) | 606  (***) | 372  (*) | 166  (NS) |
| OC2 |  |  | - | 277  (**) | 196  (NS) | 479  (*) | 475  (*) | 549  (**) | 365  (*) |
| PG |  |  |  | - | 140  (NS) | 163  (NS) | 164  (NS) | 240  (**) | 379  (*) |
| GR |  |  |  |  | - | 192  (NS) | 179  (NS) | 243  (**) | 316  (*) |
| OC3 |  |  |  |  |  | - | 249  (**) | 72  (NS) | 364  (*) |
| TO |  |  |  |  |  |  | - | 380  (*) | 753  (***) |
| HZ |  |  |  |  |  |  |  | - | 243  (*) |
| CT |  |  |  |  |  |  |  |  | - |

CTR: control diet; CR: control diet + carob; OC2: control diet + 2POC by-products; PG: control diet + pomegranate; GR: control diet + grape; OC3: control diet + 3POC by-products; TO: control diet + tomato; HZ: control diet + hazelnuts; CT: control diet + citrus. *,**,**, NS: p<0.05, p<0.001, and not significant respectively.
